# Emerging roles of inositol pyrophosphates as key modulators of fungal pathogenicity

**DOI:** 10.1080/21505594.2017.1421832

**Published:** 2018-02-27

**Authors:** Hyun Ah Kang

**Affiliations:** Department of Life Science, Chung-Ang University, Seoul, Korea

**Keywords:** Inositol pyrophosphates, Pathogenicity, Fungal infection, *Cryptococcus neoformans*

## Abstract

Inositol pyrophosphates (PP-IPs) are energy-rich small molecules that are omnipresent in eukaryotic cells, from yeast to mammals, playing central roles in overall cellular homeostasis as a diverse and multifaceted class of intracellular messengers. Recent studies of the metabolic pathways and physiological roles of PP-IPs in the human pathogenic fungus *Cryptococcus neoformans* have revealed that the PP-IP_5_ (IP_7_) is a key metabolite essential for fungal metabolic adaptation to the host environment, immune recognition, and pathogenicity. This suggests the PP-IP biosynthesis pathway, comprising phospholipase C1 (Plc1) and a series of sequentially acting inositol polyphosphate kinases (IPKs), as a new virulence-related signaling pathway in *C. neoformans*. Given that fungal species have a reduced array of the kinases required for the synthesis of PP-IPs and that the homology between human and fungal IPKs is restricted to a few catalytically important residues, identification of IPK inhibitors specifically targeting the kinases of pathogenic fungi has emerged as a desirable and achievable strategy for antifungal drug development.

Inositol pyrophosphates (PP-IPs) play essential roles in a wide range of cellular processes in eukaryotes, governing cell physiology and homeostasis. The synthesis of PP-IPs, comprising inositol, phosphate, and pyrophosphate, is mediated by a series of inositol polyphosphate kinases (IPKs) that sequentially phosphorylate inositol triphosphate (IP_3_) to generate higher phosphorylated forms [1]. The most physiologically relevant PP-IPs are IP_7_ (diphosphoinositol pentakisphosphate, PP-IP_5_) and IP_8_ (bisdiphosphoinositol tetrakisphosphate, (PP)_2_-IP_4_). These water-soluble and energy-rich small molecules are ubiquitously present in all eukaryotic cells, from yeast to mammals. Diverse cellular actions of PP-IPs have been reported, such as improvement in cell homeostasis via reduction of the energy charge, increased phosphate uptake, improvement of mitochondrial performance, and an increase of insulin secretion in mammals [1]. PP-IPs exhibit pleiotropic effects in eukaryotic cells by regulating various biological processes as a consequence of binding to or pyrophosphorylating many different proteins [1,2].

The participation of PP-IPs in multiple signaling and metabolic pathways has been intensively investigated in the budding yeast *Saccharomyces cerevisiae*. The pleiotropic effects suggest that PP-IPs are essential for normal cell growth and stress resistance in yeast. Deletion of the *S. cerevisiae KCS1* gene, encoding an inositol hexakisphosphate kinase, resulted in decreased levels of IP_7_ and IP_8_, slow growth at 30°C, temperature sensitivity at 37°C, and increased cell volume, along with decreased resistance to salt stress and cell wall integrity [3]. The *S. cerevisiae kcs1*Δ mutant also shows vacuolar abnormalities [4], reduced uptake of inorganic phosphates [5], and decreased survival compared with wild type cells by treatment of the DNA-damaging agent phleomycin [6]. In *S. cerevisiae*, PP-IPs were also reported to function in parallel with the target of rapamycin complex 1 (TORC1) pathway to regulate the class I histone deacetylase Rpd3L, thus, regulating chromatin remodeling in response to stress or starvation signals [7]. A recent study further demonstrated that RNA Pol I, the enzyme responsible for rRNA synthesis, is pyrophosphorylated by IP_7_, and that rRNA synthesis and ribosome levels are reduced in the absence of PP-IPs [2]. However, their role in fungal pathogenesis with respect to virulence has not yet been systematically addressed.

The opportunistic human fungal pathogen *Cryptococcus neoformans* is a basidiomycetous yeast of global significance that commonly infects immunocompromised hosts, including those with AIDS. *C. neoformans* is responsible for more than 1 million cases of HIV-associated cryptococcal meningitis per year worldwide and over half a million deaths each year, predominantly in sub-Saharan Africa [8,9]. The pioneer work on the physiological functions of PP-IPs in fungal pathogens was carried out by the research group of Dr. J.T. Djordjevic, which delineated the PP-IP biosynthesis pathway in *C. neoformans* using gene deletion and PP-IP profiling analysis [10–12]. They identified the cryptococcal kinases responsible for the production of PP-IPs (IP_7_/IP_8_): Arg1 was identified as an IP_3_/IP_4_ kinase, Ipk1 as an IP_5_ kinase, Kcs1 as the major IP_6_ kinase (producing IP_7_), and Asp1 as an IP_7_ kinase (producing IP_8_). All of the mutants of cryptococcal IPKs were similarly attenuated in *in vitro* virulence phenotypes, including laccase, urease, and growth under oxidative/nitrosative stress. Kcs1-derived IP_7_ was reported to be the most crucial PP-IP for cryptococcal drug susceptibility and the production of virulence determinants. Interestingly, the *KCS1* deletion strain of *C. neoformans* was unable to utilize alternative carbon sources for growth, which might be ascribed to its reduced survival in the low-glucose environment of the host lung. Despite this metabolic defect, the *kcs1*Δ mutant established persistent low-level asymptomatic pulmonary infection without eliciting a strong immune response *in vivo* and *in vitro*. Based on the transcriptome data showing the decreased mRNA levels of surface mannoproteins in the *kcs1*Δ mutant compared to the wild type, it was speculated that the reduced recognition of the *kcs1*Δ cells by monocytes might be correlated with reduced exposure of mannoproteins on the *kcs1*Δ mutant cell surface.

In a subsequent paper, published in the previous issue of *Virulence*, the same research group reports the characterization of the enzymatic activity of Arg1 as an IPK with dual specificity (IP_3_/IP_4_) to produce IP_5_ in *C. neoformans* [13]. Of note, the *arg1*Δ mutant showed more deleterious phenotypes than the downstream IPK mutant *kcs1*Δ and exhibited a higher rate of phagocytosis by human peripheral blood monocytes with more rapid clearance from the lung in a mouse model compared to *kcs1*Δ. This observation is in contrast to the findings for *kcs1*Δ, which establishes a chronic, confined lung infection. Based on these observations, the authors concluded that Arg1 is the most crucial IPK for cryptococcal virulence in animal models, conveying PP-IP_5_–dependent and novel PP-IP_5_–independent functions; while PP-IP_5_ is essential for metabolic and stress adaptation, PP-IP_5_ is dispensable for virulence-associated functions such as capsule production, cell wall organization, and normal *N*-linked mannosylation of the virulence factor phospholipase B1.

The ability of *C. neoformans* to adapt to the host environment is mediated by several key signaling pathways, including the calcineurin, mitogen-activated protein kinase/protein kinase C (Mpk1/Pkc1), cyclic adenosine monophosphate/protein kinase A (cAMP/Pka1), high osmolarity glycerol (HOG), and Rim101 pathways [14]. A series of research papers presented by the group of Dr. J.T. Djordjevic strongly support the notion that the PP-IP biosynthesis pathway, comprising phospholipase C1 (Plc1) for IP_3_ generation and a series of sequentially acting IPKs for higher phosphorylation, is a new virulence-related signaling pathway in *C. neoformans*. However, several issues remain to be addressed, such as the understanding of molecular mechanism by which the depletion of PP-IPs in the *arg1*Δ and *kcs1*Δ mutants generates defects in the utilization of diverse carbon sources and how to affect the transcription activity of cell-surface mannoproteins. Moreover, the cross-talk between IPKs with other kinases or transcription regulators involved in different signaling pathways would be a quite intriguing subject to provide deeper insight into understanding the interplay between signaling and metabolism. Future studies are also required to investigate the regulation of expression, activity, subcellular localization, and interacting partners of IPKs. This information can be linked to the protein targets through which PP-IPs influence individual pathways governing cell physiology.

Regardless of the detailed mechanisms, the findings highlight the fungal PP-IP metabolic pathway as a new drug development target. At present, besides *C. neoformans*, the metabolic pathway that leads to the synthesis of PP-IPs has been characterized in detail only in the parasite *Trypanosoma brucei*, which causes African trypanosomiasis or sleeping sickness. The analysis of IPK-null mutants of *T. brucei* also demonstrated the importance of these signaling molecules to the fitness of this organism [15]. Amino acid sequence homology analysis revealed that homology between human and microbial IPKs is restricted to a few catalytically important residues. For example, *C. neoformans* IPKs display quite low identities to mammalian homologs, ranging from only 12.65% to 19.18% [12]. Such low homology of IPKs to mammalian enzymes has also been found in other medically important opportunistic fungal pathogens, including *Candida albicans*, potentially extending the applicability of IPK inhibitors to other fungal pathogens. Moreover, unicellular organisms are reported to have a reduced array of the kinases required for the synthesis of PP-IPs, possessing only the IPMK path, while human cells possess two metabolic routes to IP_6_, the IPMK path and the ITPK1 path [16]. Based on their crucial roles in PP-IP_5_ production, inhibitors specifically directed against Arg1 and Kcs1 might be expected to emerge as desirable drug candidates that could potentially act synergistically with azoles and improve the treatment outcome of fungal infection.
